# Supplementary material for: Comparative genomics provides new insights into the diversity, physiology, and sexuality of the only industrially exploited tremellomycete: Phaffia rhodozyma
Source: BMC Genomics. 2016 Nov 9;17:901. doi: 10.1186/s12864-016-3244-7 (PMC5103461; doi:10.1186/s12864-016-3244-7)
Supplement: Additional file 6: — List of orphan genes with links to PFAM (related to Additional file 1: Table S1). (ZIP 1428 kb) [file 12864_2016_3244_MOESM6_ESM.zip › PFAM_UNIQUE/G02994_P.html]

#                                                                            --- full sequence --- -------------- this domain -------------   hmm coord   ali coord   env coord
  
# target name        accession   tlen query name           accession   qlen   E-value  score  bias   #  of  c-Evalue  i-Evalue  score  bias  from    to  from    to  from    to  acc description of target
  
#------------------- ---------- ----- -------------------- ---------- ----- --------- ------ ----- --- --- --------- --------- ------ ----- ----- ----- ----- ----- ----- ----- ---- ---------------------
  
G02994\_P             -             89 Dehydrin             PF00257.14   153     0.074   12.0   0.8   1   1    0.0001       0.1   11.6   0.7    92   121     2    26     1    66 0.82 - Dehydrin  
G02994\_P             -             89 DUF2057              PF09829.4    189      0.14   10.5   2.2   1   1   5.6e-05      0.17   10.2   2.2    98   157    15    76     6    85 0.73 - Uncharacterized protein conserved in bacteria (DUF2057)  
G02994\_P             -             89 DUF4414              PF14377.1    108    0.0098   14.5   1.3   1   1   7.6e-06     0.011   14.3   1.3    35    87    13    64     3    81 0.78 - Domain of unknown function (DUF4414)  
G02994\_P             -             89 FAM220               PF15487.1    278     0.066   11.2   1.1   1   1   5.9e-05     0.071   11.1   1.1    59   123    12    82     4    87 0.82 - FAM220 family
